# Regulatory Cells in Multiple Sclerosis: From Blood to Brain

**DOI:** 10.3390/biomedicines10020335

**Published:** 2022-02-01

**Authors:** Leticia Calahorra, Celia Camacho-Toledano, Mari Paz Serrano-Regal, María Cristina Ortega, Diego Clemente

**Affiliations:** Grupo de Neuroinmuno-Reparación, Hospital Nacional de Parapléjicos, Finca La Peraleda s/n, 45071 Toledo, Spain; lcalahorram@externas.sescam.jccm.es (L.C.); ccamachot@sescam.jccm.es (C.C.-T.); msregal@externas.sescam.jccm.es (M.P.S.-R.); mcristinao@sescam.jccm.es (M.C.O.)

**Keywords:** MDSCs, Treg, Breg, CD56^bright^, microglia, remyelination, histopathology

## Abstract

Multiple sclerosis (MS) is a chronic, autoimmune, and neurodegenerative disease of the central nervous system (CNS) that affects myelin. The etiology of MS is unclear, although a variety of environmental and genetic factors are thought to increase the risk of developing the disease. Historically, T cells were considered to be the orchestrators of MS pathogenesis, but evidence has since accumulated implicating B lymphocytes and innate immune cells in the inflammation, demyelination, and axonal damage associated with MS disease progression. However, more recently the importance of the protective role of immunoregulatory cells in MS has become increasingly evident, such as that of myeloid-derived suppressor cells (MDSCs), regulatory T (Treg) and B (Breg) cells, or CD56^bright^ natural killer cells. In this review, we will focus on how peripheral regulatory cells implicated in innate and adaptive immune responses are involved in the physiopathology of MS. Moreover, we will discuss how these cells are thought to act and contribute to MS histopathology, also addressing their promising role as promoters of successful remyelination within the CNS. Finally, we will analyze how understanding these protective mechanisms may be crucial in the search for potential therapies for MS.

## 1. Introduction

Multiple sclerosis (MS) is a chronic, immune-mediated, and multifocal demyelinating disease of the central nervous system (CNS). It is one of the most common causes of neurological disability in young adults, and to date, there is still no cure for this condition [1]. The prevalence of MS has been globally estimated to be 30.2 cases/100,000 population, an increase of 10.4% from 1990 to 2016 [2], and this represents a real challenge in the allocation of healthcare resources and planning in our ageing societies. MS can affect any part of the CNS, and thus, its clinical manifestations are often diverse, with signs and symptoms that vary widely depending on the extent and location of the damaged areas. Based on the clinical data available, MS is commonly divided into three clinical types. The first and most frequent is that of relapsing–remitting MS (RRMS), which represents 85% of MS cases at onset. RRMS is characterized by attacks (relapses) followed by periods of partial or complete recovery (remission). While distinguished by a relatively stable course of the attacks, RRMS can shift to the so-called secondary progressive MS (SPMS), in which the clinical symptoms produced by the attacks gradually deteriorate. Finally, the primary progressive form of MS (PPMS) is associated with steady neurological deterioration from onset, the symptoms of which continue to worsen without obvious phases of remission [1].

Although the precise etiology and the mechanisms underlying MS remain poorly understood, the infiltration of immune cells into the CNS in the presence of pre-existing genetic and environmental factors could lead to the development of the disease [3]. Briefly, autoreactive CD4^+^ T cells that escape negative selection and clonal deletion pass through a disrupted blood–brain barrier and enter the CNS. These activated CD4^+^ T cells lead to the recruitment of other inflammatory cells, such as microglia, macrophages, and B cells, which in turn drive the production of the antibodies and pro-inflammatory cytokines that destroy the myelin sheath [3,4]. Together with peripheral immune cells, resident CNS cells promote the chronicity of inflammation in MS, which may reflect a long-term stress response to homeostatic dysregulation in the CNS that leads to progressive and irreversible neurological deterioration. Most information on the immune mechanisms that mediate CNS lesions in the MS is derived from research using the experimental autoimmune encephalomyelitis (EAE) model, one of the best characterized and most frequently used animal models of this disease [5].

Focal inflammatory lesions with primary demyelination, axonal loss, and reactive gliosis in the white and grey matter are pathological hallmarks of MS [6]. Active lesions are characterized by a loss of myelin and the diffuse and dense infiltration of the complete lesion area with macrophages/microglia and T cells. Mixed active/inactive lesions, also referred to as chronic active lesions, are characterized by a hypocellular lesion core with a rim (or periplaque) of activated microglia/macrophages at the lesion border. Inactive lesions are also hypocellular and almost completely depleted of mature oligodendrocytes (OLs). Remyelination has been demonstrated ultrastructurally in MS tissue [7], and such areas of remyelination are frequently but not exclusively found at the border of active lesions and at the rim of mixed active/inactive lesions [8]. It is important to note that controlling the immunological insult plays an important role in efficient remyelination [9], such that a more detailed description of the influence of regulatory cells in MS histopathology is still required.

The immune response has several elements that restrain autoreactivity and inflammatory reactions, thereby avoiding uncontrolled tissue damage, restoring peripheral tolerance, and promoting remyelination. The mechanisms driving the maintenance or restoration of self-tolerance have been the object of much interest over the past decade. Thus, this review offers the reader an updated overview of the immunoregulatory mechanisms involved in the resolution of inflammation and how they break down in MS, moving from the periphery to the CNS. In addition, we will address the role of regulatory cells in remyelination, as well as their potential use as biomarkers and in personalized therapies aimed at enhancing immunoregulatory responses in pathological conditions.

## 2. Peripheral Immune Regulatory Cells in MS

Several regulatory defects have been associated with autoimmune or autoinflammatory diseases, including MS. Tolerance is the result of the clearance of self-reactive cells in the thymus and periphery, resulting in the generation of mature T and B cells that recognize foreign pathogens while exhibiting tolerance to self-antigens [10]. In this part of the review, we will summarize the role of peripheral regulatory immune cells fromboth innate and adaptive immunity in maintaining tolerance and their imbalance in MS pathology.

### 2.1. Regulatory T Cells

**Figure 1:**
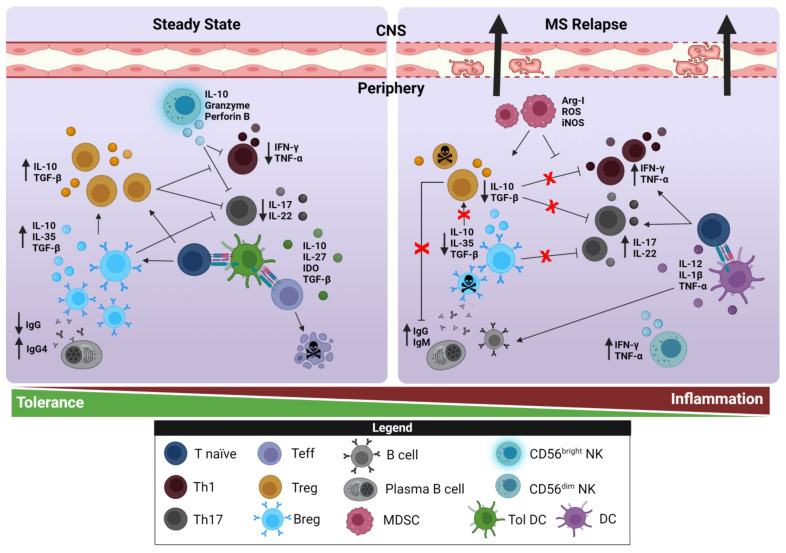
Pathogenic and protective roles of cells of the innate and adaptive immune response in steady-state conditions and during MS relapse. Alterations to or the disruption of immune regulatory mechanisms may lead to the survival and activation of autoreactive lymphocytes, which may cause and perpetuate autoimmune diseases such as MS. APCs phagocytose myelin autoantigens in the CNS released from the damaged myelin. This activates Th1 and Th17 cells, which in turn release pro-inflammatorypro-inflammatory factors such as IFN- γ, TNF-α, and IL-17. B cells secrete antibodies, and NKs and DCs release inflammatory cytokines, such as IL-12 and TNF-α. By contrast, there are several cell types from the innate and adaptive immune response (Treg, Breg, MDSC, TolDC, and CD56^bright^ NK cells) that possess a regulatory capacity, exerting protective effects. These cells have an immunoregulatory effect due to the release of anti-inflammatory cytokines, such as IL-10, IL-27, IL-35, or TGF-β, or through cell-to-cell contacts. In both cases, the action of regulatory cells inhibits the inflammatory response by reducing pro-inflammatorypro-inflammatory cytokine release, inducing T cell anergy or apoptosis. Figure created with Biorender.com (accessed on 12 January 2022).

T cells, along with B cells, form the core of the adaptive arm of the immune system. MS has long been considered a disease mediated by CD4^+^ T cells, with Th1/Th17 effector cells (Teff) considered to adopt the most prominent role in the autoimmune insult through the main cytokines they release, i.e.IFN-γ and IL-17, respectively [1]. The development of autoimmunity is fine-tuned by specific subsets of T cells, these modulating immune responses by keeping autoreactive T cells under control and maintaining a state of peripheral tolerance to a range of self-antigens [11]. However, in recent years the study of regulatory T cells (Treg) has gained importance in the physiopathology of disease. More specifically, Treg suppress the activity of various cell types, including Teff, cytotoxic CD8^+^ T cells (Tc), and antigen-presenting cells (APCs), through different mechanisms, such as cell–cell contact and the secretion of suppressive cytokines (Figure 1).

In steady-state conditions, there are two pools of Treg: natural Treg (nTreg), which originate in the thymus during development and mainly recognize self-antigens, and peripheral Treg (pTreg), which are inducible Treg that differentiate from naïve CD4^+^ T cells under specific conditions of antigen stimulation and in the presence of a particular peripheral cytokine milieu [12]. Initially, Treg were described as CD4^+^CD25^+^ cells that constitutively express the forkhead box P3 (FoxP3) transcription factor [11]. In humans, a consensus established that all circulating CD4^+^FoxP3^+^ Treg are characterized by high levels of CD25 and low levels of CD127 (CD4^+^CD25^hi^CD127^lo^FoxP3^+^) [13]. Interestingly, Treg instability has been described, and they may downregulate FoxP3 expression under inflammatory conditions and, hence, their regulatory function, thereby acquiring characteristics of Teff [14,15]. Such cells are referred to as “exTreg” (CD4^+^CD25^lo^FoxP3^lo^), and they express pro-inflammatory cytokines such as IL-17 and/or IFN-γ, losing their suppressive function while retaining the potential to become pathogenic and induce autoimmunity [16].

In MS, disruption of the Teff/Treg equilibrium may be involved in disease development, with contradictory reports regarding the frequency and/or dysfunction of blood Treg in patients [17]. Some studies found no differences in CD4^+^CD25^hi^ T cell frequency between patients and healthy controls (HCs) but showing an impaired inhibition of the CD4^+^CD25^−^ cell proliferation [18]. To shed light on this issue, a recent meta-analysis of 16 studies concluded that there was no significant difference in CD4^+^CD25^+^ T cells between MS patients and HCs, although the proportion of CD4^+^CD25^+^FoxP3^+^ Treg decreased in MS patients [19]. Along similar lines, weaker FoxP3^+^ expression was detected in children with MS in whom the ability to suppress CD4^+^CD25^−^CD127^+^ responder T cells and CD4^+^CCR2^+^CCR5^+^ Teff was worse than in HCs [20]. These data reinforce the idea that FoxP3 is a key element in the suppressive activity of Treg and that precise cell markers must be established for phenotypic analysis.

The heterogeneity of Treg may be characterized by a variety of surface markers, such as CTLA-4, CD31, CD39, CD73, and CD45RA/RO [21,22]. CD39 is an ectoenzyme that can hydrolyze ATP and ADP to AMP and impair Teff proliferation. CD39^+^ Treg are more stable in humans, with more functionality, which correlates with their ability to secrete IL-10 that inhibits proliferative responses, as well as the secretion of IL-17 and IFN-γ by Teff [23]. A CD39^+^ Treg subset could be a potential biomarker of the inflammatory activity in MS, and in this regard, a deficit in CD39^+^CD4^+^CD25^+^FoxP3^+^ Treg was seen in RRMS patients but not in SPMS patients compared with HCs. Moreover, CD39^+^ Treg of both MS patient types showed a significantly impaired capacity to suppress IL-17 production by Teff relative to HCs [24]. While stable patients have significantly fewer CD39^+^CD4^+^CD25^hi^FoxP3^+^ Treg, in samples collected during acute MS attacks, similar values to HCs were detected. Furthermore, CD25, CTLA-4, CD39, and FoxP3 mRNA expression was significantly enhanced in PBMCs from MS patients in clinical relapse relative to patients in a stable phase of the disease [25]. More recently, CD39 ATPase activity and Treg frequency increased relative to HCs in RRMS patients who underwent immunomodulatory treatment, such as IFN-β, glatiramer acetate (GA), natalizumab, or fingolimod [26,27]. Similarly to CD39, superoxide dismutase (SOD)-1, responsible for scavenging reactive oxygen species (ROS), has been recently associated with the molecular network involved in T cell response [28]. Increased intracellular amounts of SOD-1 in T cells of RRMS patients under immunomodulating therapies, except fingolimod, have been observed [29], while SOD-1 levels were reduced in RRMS individuals at disease onset [30]. Moreover, a positive correlation between SOD-1 amount and circulating Treg expressing the exon 2 of FoxP3 was observed in those RRMS patients [29]. 

Regarding the possibility that Treg provide information on the clinical course of MS, a recent study based on Helios expression (a transcription factor that defines nTreg) indicated that progressive MS patients (both PPMS and SPMS) had more pTreg (FoxP3^+^Helios^−^CD25^hi^CD127^−^) than RRMS patients. Although no differences were observed in nTreg (FoxP3^+^Helios^+^CD25^hi^CD127^−^), progressive MS patients had less FoxP3 than RRMS patients, which was more pronounced in patients with a higher expanded disability status scale (EDSS) ≥ 3.5 [31]. In studies into resting (CD45RA^+^—prior to antigen presentation) and activated Treg (CD45RA^−^—after exposure to self-antigens), the proportion of resting Treg was reduced in treated and untreated MS patients, in conjunction with a parallel increase in activated Treg [22,32]. Together these data provide indirect evidence of the potential to use Treg subsets as biomarkers of prognostic value or of disease outcomes.

Another induced Treg subset was defined as type 1 regulatory cells (Tr1), which exert their suppressive activity by secreting high levels of IL-10 and TGF-β [33]. Tr1 coexpress the CD49b, LAG-3, and CD226 surface markers, rather than CD25 and FoxP3 [34]. There are many reports of lower numbers of Tr1 in MS patients, and their failure to secrete IL-10 impairs their suppressive effects compared with HCs [35]. Coengagement of TCR and CD46 (a potent costimulator of human T cells) facilitates the conversion of Th1 T cells to Tr1, and the deficient CD46 activation observed in RRMS patients was accompanied by a loss of IL-10 secretion [36].

Along with CD4^+^ Treg, CD8^+^ Treg are also among the lymphocyte subtypes that re-cognize and lyse activated myelin-specific CD4^+^ T cells [37]. The best studied CD8^+^ Treg phenotypes in MS are CD8^+^CD25^+^FoxP3^+^ and CD8^+^CD28^−^ [38]. As with CD4^+^ Treg, a recent meta-analysis clearly showed a significant reduction in the number of CD8^+^ Treg in MS patients [39], and the frequency of circulating CD8^+^FoxP3^+^ T cells was significantly lower in the peripheral blood of MS patients during relapse than when they were in remission [40]. Interestingly, intrinsic immune regulation of neuroantigen-specific CD8^+^ Treg was observed ex vivo in HC and treatment-naive RRMS patients (quiescent MS), whereas their suppressive activity was restricted in treatment-naïve MS patients during an episode of acute exacerbation [37]. Hence, CD8^+^ Treg might be a suitable biomarker for exacerbation or disease progression in MS.

In summary, the evidence available suggests that there is a potential pathway of intrinsic immune regulation that may have implications for MS therapy. At the moment, although the data consistently indicate that Treg number/function is affected in MS patients, there is little information on the restoration of homeostasis and the resolution of inflammation through Treg-based tolerogenic therapies in MS. Indeed, more research is needed to reach conclusions on the efficacy and safety of this specific therapeutic strategy prior to its inclusion in clinical practice [41,42].

### 2.2. Regulatory B Cells

While B cells are an essential part of the humoral immune response, they can also act as specialist and highly selective APCs, as CD27^+^ memory cells, and they can generate ectopic germinal center-like structures in the CNS, as well as potentially produce both pro- and anti-inflammatory cytokines [43]. During the last two decades, the strongest direct evidence of a central role for B cells in MS autoimmunity was the demonstration that peripheral B cells were depleted by anti-CD20 therapy, leading to a rapid decrease in disease activity and a reduced number of gadolinium-enhanced lesions [44]. Interestingly, anti-CD20 therapy does not affect pro-B cells or existing plasma cells, and sera antibodies persist, unchanged after treatment, suggesting that B cells act through mechanisms other than antibody production [45]. On the other hand, B cells also play a critical role in maintaining peripheral tolerance and suppressing the development of autoimmune diseases [46,47].

The capacity of B cells to help control MS was anticipated by the fact that B cell depletion in the EAE model virtually impeded recovery after the insult [48]. Despite intense efforts, no regulatory B cell (Breg)-specific transcription factor has been identified to date, and there is no consensus on a specific phenotype of these cells. Hence, Breg are defined functionally based on their ability to suppress pro-inflammatory responses through the activity of IL-10, TGF-β, and IL-35 [49]. More specifically, Breg suppress pro-inflammatory cytokine production by dendritic cells (DCs), they support Treg expansion, and they lead to the inhibition of Teff differentiation and regulate CNS inflammation [50] (Figure 1). In humans, several Breg subsets have been identified, the most commonly described being CD24^hi^CD38^hi^ transitional B cells, CD24^hi^CD27^+^ Breg (B10 cells), and CD27^+^CD38^hi^ plasma cells/plasmablasts [47,51]. However, B cell populations can also express other regulatory molecules such as CD5, CD1d, CD25, CD71, CD73, PD-L1, or CD138 [52,53,54,55].

In recent years, the role of Breg in MS has been studied extensively, and fewer cells and functional alterations have been correlated with disease activity or severity [56]. When PBMCs are stimulated with CpG-DNA in culture, those from RRMS patients were seen to produce a lower proportion of IL-10 producing B cells compared with HCs [46]. Moreover, using CD27 to distinguish between memory (CD27^+^) and naïve (CD27^−^) B cells in humans, the proportion of naïve CD3^−^CD27^−^IL-10^+^ B cells was significantly lower in RRMS patients during relapse than those in remission. There are conflicting data regarding transitional B cells in MS patients relative to HC, and while originally no significant differences were reported, more recently, MS patients were seen to have fewer of these naïve cells, producing less IL-10 [57,58]. Conversely, the CD19^+^CD25^+^ Breg subset appears to be larger in MS patients than in HCs, and also in the relapsing as opposed to the remitting phase of the disease [54]. Interestingly, another study demonstrated that most MS patients have a specific defect at the peripheral but not central B cell tolerance checkpoint, potentially resulting in defective Treg function [59]. Accordingly, ex vivo expansion of Breg and the induction of anti-inflammatory cytokines were promoted by stimulation with CD40L [60], TLRs [61], or thymosin-α1 [62], suggesting that reduced IL-10 production in B cells may be caused by a defect in the relationship between B cells and other leukocytes, and that there is no intrinsic defect in naïve B cell production. 

As we have mentioned above, there is no doubt about the positive effect of anti-CD20 in disease activity, but other disease-modifying therapies (DMTs) affect the B cell compartment in an MS individual, which may contribute to their efficacy. More precisely, patients treated with GA, fingolimod, IFN-β, or dimethyl fumarate (DMF) showed a higher number of Breg with a restored production of IL-10 and a shift toward a more anti-inflammatory immune profile compensating the tolerogenic loss in MS [27,62,63].

### 2.3. Natural Killer Cells

Natural killer cells (NKs) are lymphoid cells that constitute part of the innate immune response and that have received considerable attention in recent years. Two functional subsets of NKs have been described in human peripheral blood: CD56^dim^CD16^+^ NKs (CD56^dim^), the most preponderant and with strong cytotoxic activity, and CD56^bright^CD16^−^ NKs (CD56^bright^) with stronger cytokine-producing capabilities [64] and a greater migratory capacity, constituting a large proportion of the NKs in the lymph nodes and cerebrospinal fluid (CSF) of MS subjects [65]. CD56^bright^ NKs might mediate T cell responses by secreting cytokines such as IFN-γ, IL-10, IL-13, TNF-α, and GM-CSF [66], which gives them regulatory properties. A more specific CD56^bright^ NK regulatory phenotype has been recently defined by the expression of CD34^−^CD117^lo^CD94^+^NKG2A^+^ [67].

In HC, CD56^bright^ NKs suppress the proliferation of autologous T cells [68] (Figure 1), and there are data pointing to a compromised immunoregulatory function of these NKs in untreated MS patients due to an exacerbated resistance of T cells to immunosuppression [69]. There is evidence that a deficiency in the cytotoxic capacity of CD56^bright^ NKs in MS patients correlates with MS attacks and with the development of new inflammatory lesions [70,71], even though CD56^bright^ NK frequencies in untreated MS patients are similar to those in HCs, but they expand in response to certain treatments, such as DMF or IFN-β [68,72].

NKs have the ability to fine-tune their activity by expressing activating natural cytotoxicity receptors (NKp30, NKp44, and NKp46), activating and inhibitory CD94/NKG2 receptors, or inhibitory KIR receptors (killer Ig-like receptors) [66]. In fact, the combinations of these receptors define the developmental stage and activity status of these cells. Different studies have explored the role of NK receptors and their HLA ligands in MS pathology, and HLA-E upregulation in T cells (a ligand for the inhibitory NKG2A receptor) has been observed in MS patients, blocking the immunosuppression by CD56^bright^ NKs [69]. The NKG2D receptor has been implicated in the generation of peripheral tolerance through the downregulation of NKG2D ligands [73]. In this regard, lower frequencies in NKG2D^+^CD3^−^CD56^+^ NKs were found in clinically isolated syndrome (CIS, i.e., MS patients at their first relapse that can derive or not in a clinically defined MS) and untreated RRMS patients than in IFN-β-treated RRMS patients, which was linked to a negative correlation between NKG2D expression and EDSS values in MS patients, treated and untreated [72].

Recently, a randomized, double-blind, placebo-controlled clinical trial (STAyCIS) drew an association between the CD8^+^ NK population (NK8^+^) and favorable clinical outcomes in RRMS patients. This new NK8^+^ subgroup had enhanced expression of the NKG2D activating receptor and reduced expression of the ILT2 and KIR2DL4 KIR receptors [74]. Indeed, NK8^+^ may downregulate autologous CD4^+^ T cells by reducing HLA-G sensitivity, regarded as a regulatory mechanism in several autoimmune diseases, including MS [75].

In summary, there is evidence indicating that NKs have immunoregulatory capabilities that are compromised in MS patients. Moreover, augmenting these cells through different DMTs such as IFN-β, DMF, or alemtuzumab [27] and restoring their immunoregulatory activity is associated with weaker inflammatory responses and disease amelioration, which might point to NKs as future biomarkers of treatment efficacy in MS.

### 2.4. Tolerogenic Dendritic Cells

DCs are a heterogeneous group of APCs with an important role in MS. DC precursors home to sites of potential antigen entry where they differentiate into immature DCs (imDCs), which are predominantly found in peripheral tissues where they act as sentinels. When they present an antigen linked to their major histocompatibility complex (MHC), these cells become mature DCs (mDCs) in which MHC-II expression is enhanced and costimulatory molecules are upregulated, such as CD40, CD80, CD86, and the chemokine receptor CCR7 [76]. At this moment, mDCs prime naive T cells to become Teff through interactions of MHC-II and the costimulatory molecules with different ligands at the T cell membrane [77] (Figure 1).

Nevertheless, DCs not only possess exceptional antigen-presenting capacity, but they also exhibit inherent plasticity, which facilitates both immunogenic and tolerogenic immune response [78]. Depending on the context in which the antigen is taken up (i.e., in steady state), DCs may induce tolerance (Figure 1). In fact, imDCs have the ability to establish and maintain tolerance as semimature DCs. These tolerogenic DCs (TolDCs) express low levels of costimulatory molecules to induce T cell proliferation, and they are more capable of releasing IL-10 [79]. TolDCs provoke passive tolerance through negative selection of autoreactive T cells (anergy) and generation of Treg de novo [80] (Figure 1). Accordingly, a new subset of DCs were identified with a high capacity to produce IL-10 in vivo (DC-10), and that promotes Tr1 differentiation via the IL-10-dependent ILT4/HLA-G pathway [79]. This discovery offers a new perspective for the treatment of autoimmune diseases. DC-10 cells have been more precisely defined as CD1a^−^CD1c^−^CD14^+^CD16^+^CD11c^+^CD11b^+^HLA-DR^+^CD83^+^, and interestingly, they express high levels of CD40 and CD86, as well as upregulate CD80 upon activation [81]. For this reason, it has been proposed that immunogenic DCs and TolDCs may best be defined based on their functional effector properties rather than their immunophenotype. This dual immunogenic or tolerogenic functionality of DCs is a double-edged sword, as a delicate balance must be maintained that, once disturbed, may lead to the development of an autoimmune disease.

For MS, DC analysis pointed to their implication in both the initiation and development of the disease [82]. The number of circulating DCs isolated from MS patients is elevated, producing higher levels of pro-inflammatory cytokines, more strongly expressing markers of activation (CD40, CD80, CD86, HLA-DR) without upregulating PD-L1, and with a greater migratory capacity [82,83]. TolDCs can be induced by numerous immunosuppressive agents, such as TGF-β, IFN- β, corticosteroids, estrogen, and neurotransmitters, the most often used being Vitamin D3 [27,84]. VitD3-TolDCs generated in vitro from monocytes behave similarly in RRMS and HC [85], making them promising candidates for specific cellular therapy. The potential of using TolDCs in therapeutic strategies for MS patients has been explored [42,86], and in two coordinated phase 1b trials, elevated IL-10 from peptide-specific T cells was associated with vitD3-TolDC therapy when loaded with a cocktail of myelin peptides (PLP, MOG, and MBP), producing an increase in the Tr1 frequency in MS patients [10,42,83]. These therapies appear to be safe and well tolerated, and they offer insights into the potential of using TolDCs to treat MS and other autoimmune diseases.

A recent in vitro study analyzed the efficacy of a combined therapy based on the use of vitD3-TolDC together with IFN-β in PBMCs from MS individuals whose results showed enhanced suppressive ability of vitD3-TolDC, representing a promising strategy for MS patients [87]. 

### 2.5. Myeloid-Derived Suppressor Cells

Myeloid-derived suppressor cells (MDSCs) are a heterogeneous population of immature myeloid cells generated in the bone marrow during myelopoiesis. In steady-state conditions, these cells mature towards circulating leukocytes (i.e., granulocytes, monocytes, and DCs) to undertake surveillance of the tissue microenvironment [88]. However, several pathological circumstances lead to a blockade of IMC differentiation, such as inflammatory insults, arresting these cells in an immature state and driving them to become functionally active MDSCs [89]. It is challenging to classify MDSCs due to their wide heterogeneity, although in mice they have been classified into two subsets based on the expression of the Ly-6G and Ly-6C epitopes of the Gr-1 antigen: monocytic-MDSCs (M-MDSCs) have a CD11b^+^Ly-6G^−^Ly-6C^hi^ phenotype, whereas polymorphonuclear-MDSCs (PMN-MDSCs) were classified as CD11b^+^Ly-6G^+^Ly-6C^lo^ cells [90]. Interestingly, these two main subpopulations have also been described among human PBMCs but with a different immunophenotype: CD11b^+^CD14^+^CD15^−^CD33^+^HLA^−^DR^−/low^ for M-MDSCs and CD11b^+^CD14^−^CD15^+^CD33^+^HLA-DR^−/low^LOX1^+^ for PMN-MDSCs [91,92].

MDSCs are characterized by their ability to impair ongoing immune responses through different mechanisms. Murine PMN-MDSCs mainly achieve this through the release of ROS, whereas murine M-MDSCs upregulate arginase-I (Arg-I) or inducible nitric oxide synthase (iNOS), and they drive the release of a huge array of immunosuppressive cytokines [93]. Unlike their mouse counterparts, both human MDSC subsets can exert immunosuppressive effects through ROS [94]. In functional terms, Arg-I and iNOS metabolize the essential amino acid L-arginine in the microenvironment, which dampens the expression of the ζ subunit of CD3 by T cells, inducing their anergy. However, iNOS can generate nitric oxide, which inhibits MHC-II expression and induces T cell apoptosis. Moreover, Treg induction, the regulation of T cell activation, the expression of negative immune checkpoint molecules such as PD-L1, and the blockade of lymphocyte homing are all alternative mechanisms of action for murine MDSCs [95].

Currently, there have been very few and controversial studies into the role of MDSCs in MS. In terms of MDSC abundance, active RRMS patients were seen to have a higher frequency of PMN-MDSCs than stable RRMS patients or HC [96]. Similar results were later obtained, additionally showing an increase in M-MDSCs during relapses but a relative decline of both cell subpopulations in SPMS patients [97]. Furthermore, fewer circulating M-MDSCs were identified in RRMS patients relative to HCs, whereas differences in PMN-MDSC abundance were not found [98]. Therefore, further longitudinal, well-designed follow-up studies are needed to adequately address the association of each MDSC subset with the clinical manifestations of MS. 

Most data regarding MDSC functionality come from the EAE model of MS. In the case of M-MDSCs, also called inflammatory monocytes or Ly-6C^hi^ cells, there are conflicting results due to their high levels of plasticity during the rapid immunological changes in EAE. A gradual expansion of Ly-6C^hi^ cells was reported after immunization in the bone marrow, blood, and spleen [99,100,101]. In fact, the enrichment of circulating CD11b^+^Ly-6G^−^Ly-6C^hi^ cells was associated with an earlier onset and increased severity of the EAE clinical course [100]. Moreover, a pronounced decrease in CCR2^+^Ly-6C^hi^ monocytes in CCR2^−/−^ mice at EAE onset was associated with the development of a milder clinical course [101]. However, the timing for M-MDSC analysis during the course of EAE must be considered to understand their true functional consequences. For example, while cells with the M-MDSC phenotype behave as professional APCs at EAE onset, at the peak of the disease, they clearly suppress T cell activity [102]. In fact, we showed that the induction of MDSC differentiation with Am80 around the peak of the clinical course of EAE impaired the immunomodulatory function of MDSCs, and consequently, it led to an increase in T cell density and compromised EAE recovery [103]. In addition, we also described that the splenic abundance of MDSCs at the peak of the disease is related to the presence of a less prominent lymphoid subset and a milder disease course, supporting their protective role [104]. Lastly, at least 50% of the anti-inflammatory M-MDSCs present in the CNS at the peak of EAE were recently demonstrated to be derived from pro-inflammatory Ly-6C^hi^ cells that colonized this organ at EAE onset [105]. Therefore, the regulatory role of M-MDSCs is clear, but the time when immunomodulation occurs should be explored (e.g., around the peak of EAE). Conversely, the regulation of CNS autoimmune inflammation due to PMN-MDSC activity has been poorly addressed. The adoptive transfer of splenic PMN-MDSCs into syngeneic recipient EAE mice during the asymptomatic period ameliorated EAE, specifically in terms of delaying disease onset, reducing the size of inflammatory lesions and producing less demyelination in the spinal cord (SC), consistent with the significantly reduced expansion of autoreactive Teff [96]. Importantly, the relationship between PMN-MDSCs not only with T cells but also with B cells has been gaining interest in recent years. In this sense, a crucial role of PMN-MDSCs has been described in controlling B cell number and activation state in the CNS of MOG-immunized EAE mice during the recovery phase [91]. Interestingly, B cells also appear to drive neutrophils to acquire a PMN-MDSC phenotype in the nervous parenchyma of EAE mice, a phenomenon that requires more profound analysis prior to its translation to humans.

Data regarding MDSC function in MS patients is scarce and incomplete. The increase in PMN-MDSCs seen in RRMS patients during relapse correlates well with their clear suppressive activity over autologous T cells [96] (Figure 1). A decrease in *STAT3* and *ARG1* expression in peripheral blood M-MDSCs from RRMS patients suggested they had weaker suppressive activity [98]. However, later M-MDSCs from RRMS patients at relapse or during remission displayed well-conserved immunosuppressive activity over activated T cells [97]. Strikingly, an analysis of cell surface proteins and of the genes expressed by M-MDSCs from SPMS patients revealed weaker IL-10 mRNA expression, and these cells promoted autologous T cell proliferation, highlighting their altered immunosuppressive activity during the progressive phase of the disease [97].

There are few reports about the effect of approved MS therapies on the number and activity of MDSCs. In EAE, we showed that M-MDSCs were more abundant and presented stronger immunosuppression after IFN-β treatment [106]. In MS patients, this has been addressed in only two studies, one of which detected a non-significant increase in the number of total M-MDSCs in RRMS patients treated with GA [98]. In the other, when human MDSCs were expanded by glucocorticoid (GlC) treatment during relapse [107], RRMS patients produced a higher proportion of PMN-MDSCs after GlC infusion that translated into higher serum Arg-1 levels, weaker expression of the β subunit of the human GlC receptor, and higher activating transcription factor 3 serum levels, which is implicated in important signaling pathways that mediate PMN-MDSC accumulation. The fact that alleviating MS with GlCs may be paralleled by the accumulation of PMN-MDSCs points to a determinant role of MDSCs in disease severity. This is supported by studies into EAE and other animal models of MS indicating that: (1) the differentiation of M-MDSCs deteriorates the clinical course of EAE [103]; (2) the potentiation of M-MDSC number and activity with cannabinoids during the induction period of a viral model of PPMS, Theiler’s murine encephalomyelitis virus-induced demyelinating disease (TMEV-IDD, a model in which chronic encephalomyelitis is induced by direct intracerebral infection of the animals with the virus), averts excessive inflammation and alleviates the motor deficits during the demyelinating phase of the disease [108]; and (3) the abundance of M-MDSCs at the peak of EAE is inversely correlated with a milder disease courses [104]. In MS, one study linked the maintenance of a high level of PMN-MDSCs in the peripheral blood of RRMS patients to the efficient control of inflammation, reaching the so-called “no evidence of disease activity” state (NEDA-3) compared with those with an ongoing disease activity [91].

In summary, while further studies are needed to better understand the role of MDSCs in the true context of MS, the aforementioned data clearly highlight the extraordinary potential of these regulatory cells as targets for therapeutic applications in this and other autoimmune diseases.

## 3. Regulatory Cells in the Brain and CSF

The relevance of regulatory cells in controlling the activation of autoreactive T cells in the context of EAE has been described not only in the periphery but also in the target organ, the CNS [109,110,111]. However, what is currently known about the presence of regulatory cells in the human CNS is limited, and there is some controversy regarding the distribution of this cell population in MS lesions. In this part of the review, we will summarize the data available on the distribution and role of regulatory cells in the CNS, focusing on results from demyelinating lesions and the CSF of MS patients.

### 3.1. Lymphoid Regulatory Cells

The defects in Treg number and function at specific sites of local inflammation have remained a matter of debate in the context of autoimmune diseases given that regulatory mechanisms should act not only at the periphery but also to control the inflammatory response at the site of local damage and allow myelin repair to occur. Given that Treg are required to re-establish CNS homeostasis once tolerance is broken [110], it seems relevant to evaluate whether regulatory cells can migrate out from the blood towards the CNS, and whether they can control the immune response locally in the nervous tissue of MS patients.

The frequency of CD4^+^CD25^hi^ Treg among the CD4^+^ T cells in the CSF was similar to that in the peripheral blood of RRMS patients [112]. However, when the FoxP3 marker was also considered for Treg identification, the abundance of CD4^+^CD25^+^FoxP3^+^ T cells was higher in the CSF than in the peripheral blood from CIS, RRMS, and SPMS patients [113,114]. By contrast, there were fewer CD8^+^ Treg (identified as CD8^+^CD25^+^FoxP3^+^ cells) in the CSF than in the peripheral blood of MS patients in relapse [115], suggesting a decrease in these regulatory cells at sites of local inflammation. 

As we mentioned earlier, SOD-1 has been recently associated in T cells with increased Treg differentiation/activity [29]. Higher levels of SOD-1 were detected in the CSF of RRMS patients compared with HC, suggesting an impaired SOD-1 secretion and augmenting ROS that could have a role in neuroimmune regulatory responses and in the pathogenesis of MS [30].

Regarding the presence of Treg in MS tissue, specific HLA-G^+^ nTreg were described in the perivascular areas of active MS lesions [116], supporting the idea that the recruitment of regulatory cells to the CNS may also help directly control immune responses at the sites of inflammation in pathological conditions. By contrast, the presence of FoxP3^+^ cells was not initially detected at any type of MS lesions [117], and subsequent analysis of the distribution of FoxP3^+^ cells in MS tissue confirmed the scarcity of these regulatory cells, with FoxP3 staining only witnessed in 70% of patients [118,119]. Although the presence of FoxP3^+^ Treg has been observed in all types of MS lesions, 50% of them within the active region and periplaque of chronic active lesions have a more relevant immunoregulatory phenotype based on IL-10 expression [120]. The lack or scarcity of Treg in MS tissue might be related to the fact that this regulatory cell type is considered an extremely short-lived population [121], consistent with the increase in apoptotic Treg (CD4^+^CD25^hi^CD127^lo^CD95^hi^CD45RO^hi^) in the CSF relative to the peripheral blood [118].

Interestingly, it is well established that the migration and infiltration of Treg into their target tissue is mainly controlled by specific chemokine receptors [122,123]. In this sense, the accumulation of HLA-G^+^ nTreg cells in the CSF may be due to CCR5-mediated transmigration [116]. Given the relevance of the migratory capacity of Treg to enrich the CNS, the possible effects of the drugs available for MS treatment on this cell population should be taken into account. Although Foxp3^+^ Treg have low levels of CD49d (i.e., the alpha-4 chain of VLA-4), treatment with the monoclonal antibody natalizumab blocks the transmigration of FoxP3^+^ Treg in a similar manner to FoxP3^−^CD4^+^ nonregulatory T cells isolated from the peripheral blood of MS patients [124]. Despite modulating the migration of Treg, the beneficial therapeutic effect of natalizumab persists, so the biological significance of disturbing Treg transmigration after MS treatment remains unclear.

**Figure 2:**
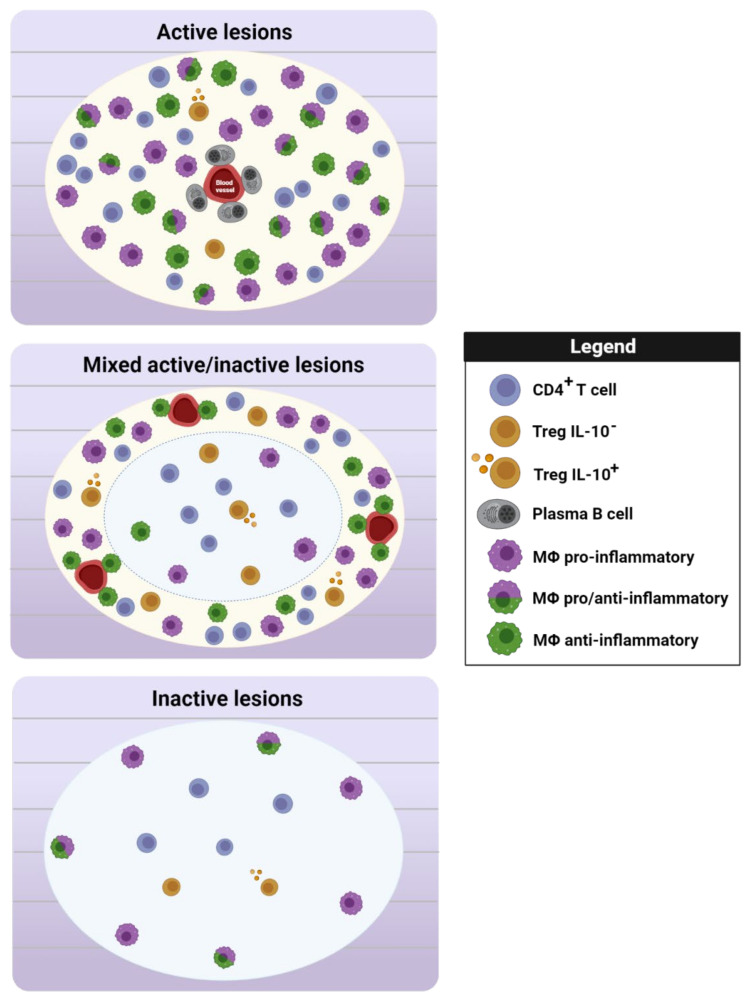
Scheme of the regulatory cells present in MS lesions. The IL-10^+^ Treg/Treg ratio increases in the plaque of active and inactive lesions and at the rim of mixed active/inactive areas (Zandee et al., 2017). Perivascular CD138^+^CD38^+^ plasma cells act as a source of the anti-inflammatory cytokine IL-10, and they are mainly observed in active lesions (Machado-Santos et al., 2018). In terms of myeloid cells, the distribution of typical markers used to identify pro- or anti-inflammatory cells in demyelinating lesions reveals the presence of myeloid cells with an intermediate activation phenotype (CD40^+^/CD206^+^ cells) in active lesions (Vogel et al., 2013) and the coexistence of pro-inflammatory and regulatory myeloid cells at the rim of both mixed active/inactive and inactive lesions (Vogel et al., 2013; Jackle et al., 2020). However, anti-inflammatory myeloid cells are also observed in active lesions and at the rim of mixed active/inactive lesions (Miron et al., 2013). Legend: CD4^+^ T cell = CD4^+^ FoxP3^−^; Treg IL-10^−^ = CD4^+^ FoxP3^+^IL-10^−^; Treg IL-10^+^ = CD4^+^ FoxP3^+^ IL-10^+^; plasma B cell = CD138^+^CD38^+^; Mϕ pro-inflammatory = iNOS^+^ or CD40^+^ cells; Mϕ pro-/anti-inflammatory = CD40^+^CD206^+^ cells; Mϕ anti-inflammatory = CD206^+^ or CD163^+^ cells. Figure created with Biorender.com (accessed on 12 January 2022).

There is little evidence that Treg present in the CSF maintain their regulatory function. However, the alternative population of HLA-G^+^ nTreg isolated from the CSF of MS patients was able to inhibit the proliferation of T cells in response to a polyclonal stimulation [116]. Alternatively, CD39^+^ Treg have been recently characterized in the CSF of RRMS at relapse, and a direct correlation between the levels of CD39 mRNA and the expression of IL-10 in the CSF was established in these patients, indicating that Treg from the CSF can maintain some regulatory activity [125]. In parallel, IL-10^+^ Treg have been described within demyelinating lesions, suggesting that Treg might also maintain their suppressor activity within MS tissue and not only in the CSF [120] (Figure 2). In murine models, the presence of the IL-33 receptor (ST2) has been associated with highly regulatory cells [126]. In this sense, ST2 expression was observed in 60% of FoxP3^+^ cells in active lesions, while the expression of this receptor was virtually absent from chronic lesions. These data suggest that Treg may be activated by the inflammatory environment, and their suppressor activity might be greatest in those areas prone to be repaired [120].

Much less data are available about the presence and role of Breg within the CSF or CNS of MS patients. The proportion of CD19^+^CD25^+^ Breg was seen to be higher in the CSF than in the peripheral blood of CIS patients during relapse, with a stronger presence of Breg also coexpressing FoxP3 and perforin [54]. Interestingly, the distribution and phenotype of the B cell lineage were characterized recently in human tissue, reflecting the higher density of perivascular CD20^+^ B cells in patients with acute MS than during the progressive disease course [127]. On the other hand, the number of plasma cells (identified as CD138^+^ cells) and the plasma cell/B cell ratio were higher in active lesions from progressive MS patients (Figure 2), suggesting that infiltrating B cells could gradually differentiate into plasma cells during the disease course. Given that plasma cells are considered to be a prominent source of IL-10 within the CNS [128], this B cell population in MS tissue could be considered a source of potent regulatory cells due to the secretion of this anti-inflammatory cytokine.

There are little data about the role of CD56^bright^ NKs in the CSF or CNS of MS patients. However, an extensive study into the phenotype of immune cells in the CSF showed that CD56^bright^ NKs were enriched in the CSF of all types of MS, as well as in patients with other immunological diseases, suggesting that a higher abundance of these regulatory NKs may not be MS specific [129]. Whether the function of CD56^bright^ NKs from the CNS is altered in MS remains unclear. Given that NK cytotoxicity was reported to be weaker in the presence of HLA-E in CSF and MS lesions [130], CD56^bright^ NK immunoregulatory function might also be compromised in the context of MS tissue, as observed in the periphery.

### 3.2. Myeloid Regulatory Cells

In addition to the crucial role played by the regulatory cells that contribute to the adaptive immune response in controlling inflammation in MS, myeloid cells can also exert beneficial effects, and they are more abundant than lymphocytes in all types of MS lesions [6]. It is well established that microglia/macrophages can polarize in response to different cytokines and switch their phenotype from a classically activated or pro-inflammatory status to an alternatively activated or anti-inflammatory/regulatory state [131]. Although alternatively activated microglia/macrophages have been attributed a relevant role as immune response regulators that produce anti-inflammatory cytokines such as IL-10, IL-4, and IL-13 [132], it remains unclear how they might enter this activation state and influence lesion development in MS tissue. The distribution of pro- or anti-inflammatory markers in MS tissue reveals the predominance of myeloid cells with an intermediate activation status, as defined by the coexpression of CD40 (typical of pro-inflammatory APCs) and the mannose receptor (CD206, typical of anti-inflammatory macrophages) at active lesions [133]. The coexistence of iNOS^+^ or CD40^+^ cells along with microglia/macrophages expressing the anti-inflammatory marker CD163 [134] has also been observed at the rim of slowly expanding lesions (known as mixed active/chronic active lesions), supporting the absence of a well-defined pro- or anti-inflammatory phenotype for microglia/macrophages in such MS lesions [135]. Alternatively, a higher abundance of CD206-expressing macrophages has been described, mainly in active lesions and at the rim of chronic active lesions, areas where the resolution of inflammation by regulatory cells plays a crucial role in avoiding a chronic inflammatory environment [9]. However, it remains to be determined whether the decrease in regulatory cells at remyelinating lesions helps achieve effective remyelination, particularly given the scarcity of CD206-expressing macrophages and Treg in shadow plaques [9,120]. Interestingly, IL-9 may regulate the activation of macrophages in the brain of MS patients [136]. Apart from the inverse correlation between the number of activated CD68^+^ macrophages and IL-9^+^ cells in demyelinating lesions, human macrophages were seen to increase TGF-β production due to IL-9 stimulation in vitro, suggesting that IL-9 fulfills a protective role by modulating the inflammatory properties of these cells. Hence, the data available suggest that a shift in the phenotype of macrophages towards a beneficial regulatory phenotype would be an attractive therapy for MS.

Among the innate immune cells, MDSCs represent a potentially interesting subpopulation that has gained relevance in relation to controlling immune responses. As mentioned earlier, the distribution and role of MDSCs in the context of MS has been mainly assessed in the peripheral blood of patients, with controversial results [96,97,98]. In the only study describing the presence of PMN-MDSCs in the CSF of MS patients [91], an inverse correlation was found between CD138^+^ B cells and PMN-MDSCs in this body fluid, suggesting a positive role of the latter through the prevention of B cell invasion of the nervous system. Although the presence of M-MDSCs in the CNS of MS patients remains unclear, the role of these cells has been analyzed in nervous tissue from EAE mice. Both PMN-MDSCs and M-MDSCs are found in the inflammatory infiltrates of the SC at the peak of EAE, although some reports, including previous work from our group, demonstrated that M-MDSCs (identified as CD11b^+^Gr1^+^CD115^+^Arg-I^+^ cells) are the main MDSC subset in this animal model [99,102,111]. In accordance with a role of M-MDSCs in suppressing T cell function during the transition from the peak to the chronic phase of the disease [99], we found that the presence and density of M-MDSCs and the proportion of apoptotic T cells were correlated with the time course of EAE [111]. Furthermore, we showed a direct correlation between M-MDSC abundance and the degree of T cell apoptosis within the inflamed CNS [104], which is maximal when MDSCs remain in an immature state [103]. Indeed, their polarization towards mature myeloid phenotypes observed in vivo exacerbates the clinical status of EAE mice [103].

In line with these results, and given the lack of information about the role of MDSCs in the local resolution of inflammation in the CNS of MS patients, these cells represent a promising population to study in order to better understand the regulatory mechanisms in MS pathology.

## 4. The Role of Regulatory Cells in Remyelination

In MS, characteristic inflammatory activity harms OLs and destroys the myelin sheath that surrounds axons in the CNS, a process known as demyelination [137]. These events result in impaired action potential propagation, and they deprive axons of the metabolic and trophic support they receive from OLs [138]. As a result, endogenous myelin regeneration (i.e., remyelination) is crucial to not only restore axon structure and function but also prevent axonal degeneration [137,139], which should therefore limit the disability experienced by MS patients. In the CNS, remyelination is mostly carried out by oligodendrocyte precursor cells (OPCs), although recent studies point to surviving mature OLs as potentially contributing to this phenomenon [140,141]. Remarkably, Schwann cells can also participate in remyelination, especially those originating in the CNS [142]. This highly orchestrated process—involving both extrinsic and intrinsic factors—is sequentially divided into three different phases: the activation and recruitment of OPCs to lesion areas, the differentiation of OPCs into mature myelinating OLs, and the subsequent ensheathing of demyelinated axons [143]. Improving remyelination currently represents a major therapeutic challenge, and it has been the focus of many clinical trials, with limited results [144]. For this reason, and in the context of an inflammatory disease such as MS, the search for new therapeutic strategies to enhance remyelination by promoting endogenous regulatory systems is gaining importance. In addition to the positive effects of both cells of the innate and adaptive immune response in the pathogenesis of MS and EAE [145], they may also influence remyelination. Thus, in this part of the review, we will summarize how microglia/macrophages, Treg, Breg, and MDSCs may influence remyelination.

### 4.1. Microglia/Macrophages

**Figure 3:**
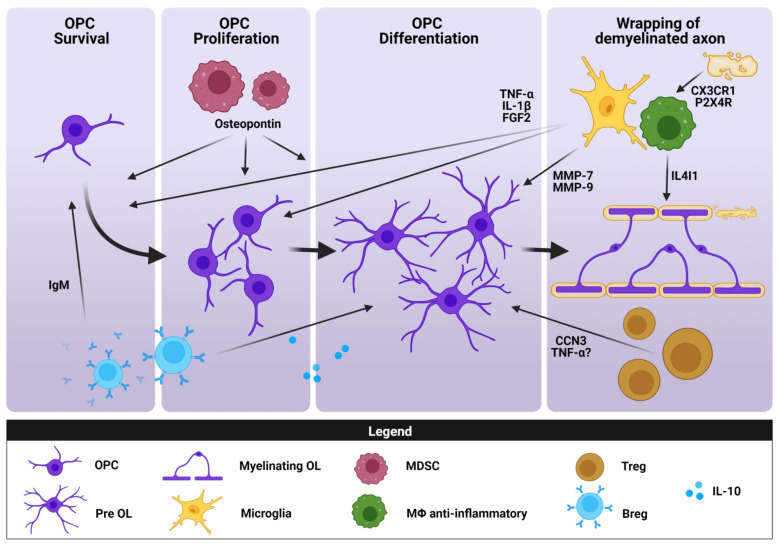
The role of immune regulatory cells in promoting remyelination. Immune regulatory cells contribute to the different phases of remyelination (OPC survival, proliferation, and differentiation towards mature OLs) by removing myelin debris (microglia/macrophages) or secreting various active molecules (microglia/macrophages, MDSCs, Treg) or even antibodies (Breg). Figure created with Biorender.com (accessed on 12 January 2022).

Microglia and infiltrated monocyte-derived macrophages survey the CNS microenvironment for signs of infection or damage. As mentioned above, they adopt distinct activation states ranging from the classically activated or pro-inflammatory to the alternatively activated or anti-inflammatory/regulatory [131]. Although microglia/macrophages have traditionally been implicated in exacerbating inflammation by secreting toxic molecules [146,147], they also contribute to myelin regeneration by: (1) phagocytosis of apoptotic cells and myelin debris, (2) the secretion of factors that influence OPC behavior [148,149], and (3) extracellular matrix remodeling (Figure 3) [150,151]. During remyelination, these cells undergo a transition from the pro-inflammatory state in the OPC recruitment phase to an immunoregulatory one in the OPC differentiation phase. In MS tissue, mannose receptor (CD206^+^) anti-inflammatory microglia/macrophages are more abundant at active lesions and in the periplaque of chronic active lesions with ongoing remyelination than at the center of chronic active or chronic inactive lesions where remyelination does not occur [9]. Hence, these microglia/macrophages are probably required to resolve inflammation, but they may leave such sites to favor remyelination. Defects in myelin debris removal from the extracellular space impair remyelination by inhibiting OPC differentiation [152,153]. Thus, blocking myelin phagocytosis in mice lacking the fractalkine receptor CX3CR1 (CX3CR1^−/−^) that are subjected to cuprizone-induced demyelination (a toxin-induced model of demyelination with no signs of lymphocyte infiltration but in which microgliosis and astrogliosis occur [144]) results in abnormal remyelination, as witnessed by the effects on myelin sheath and axonal integrity. Microglia in CX3CR1^−/−^ mice do not differ from those in wild-type (WT) mice in terms of their inflammatory responses. However, they displayed decreased levels of expression of the triggering receptor expressed on myeloid cells 2 (TREM2) gene, which encodes a protein involved in phagocytosis, and they lack endosomes, internalized myelin, and cholesterol crystals, suggesting less phagocytic activity [154]. Moreover, clearance of myelin debris is impaired in cuprizone-demyelinated TREM2-deficient (TREM2^−/−^) mice, which develop a stronger axonal pathology and clinical deficits. These mice have fewer microglia in demyelinated areas, which also have a resting morphology, lower expression levels of MHC-II and iNOS markers of activation than in WT mice [155]. This reduction in microglial activation is interpreted as a defective response to myelin damage, which is associated with the stronger pathology in the cuprizone-demyelinated TREM2^−/−^ mice, highlighting the involvement of TREM2 in microglial activation. While apparently contradictory, the reduction in the classical pro-inflammatory markers in TREM2^−/−^ mice is clearly associated with poor remyelinating activity. As indicated for TolDCs, this could be additional evidence that the presence of classic pro-inflammatory markers such as iNOS or MHC-II is not always indicative of deleterious activity of regulatory cells. Therefore, the activity rather than the phenotype of regulatory cells in neuroinflammatory contexts should be studied further.

Strong expression of the ionotropic purinergic receptor P2X4 (P2X4R) has been seen in activated microglia, both in human MS optic nerve samples and in EAE [156]. P2X4R signaling was recently highlighted as an important modulator of remyelination [157], and its potentiation in vivo by the allosteric modulator ivermectin improves motor deficits in EAE and electric nerve conduction. Moreover, in vitro P2X4R signaling induces the anti-inflammatory phenotype of microglia, evident through stronger expression of the MRC1 mannose receptor, and of the anti-inflammatory genes Arg1 and Mrc1, coupled to weaker expression of the pro-inflammatory genes Ccl2 and Nos2. Moreover, ivermectin enhances the degradation of myelin debris. Finally, ivermectin favors remyelination in organotypic cerebellar slices in the lysolecithin (LPC)-induced model of demyelination [157], another well-characterized model of demyelination induced by a toxin that has been used to study remyelination in vitro with minor participation of the adaptive immune responses.

Besides their phagocytic activity, activated microglia/macrophages secrete various molecules that directly or indirectly promote remyelination (Figure 3). On the one hand, growth factors such as fibroblast growth factor 2 (FGF-2), insulin-like growth factor 1 (IGF-1), and brain-derived neurotrophic factor (BDNF) or molecular signals such as semaphorin 3F exert an important influence on OPC migration, differentiation, and myelination [158,159,160,161,162]. In active MS lesions and in the periplaque of chronic active lesions where remyelination normally occurs, activated microglia/macrophages upregulate FGF-2, probably inducing the recruitment of FGF receptor 1 expressing (FGFR1)^+^ OPCs and prompting remyelination. By contrast, the macrophagic cells associated with lesions where remyelination comes to an end (shadow plaques) have less FGF-2-expressing cells [160]. This is consistent with what is mentioned above about the scarcity of CD206-expressing macrophages and Treg in such lesions [9,120]. BDNF has also been detected in microglia/macrophages and in T cells from active and inactive MS lesions, while the BDNF receptor gp145TrkB was observed in neurons in the surroundings of these plaques [163]. A more recent study showed that BDNF enhances OL myelination in vitro. In particular, BDNF may promote the differentiation of premyelinating OLs into myelinating ones, an effect that seems to be dependent on the presence of neurons [162]. Hence, BDNF secreted by regulatory immune cells might indirectly exert a pro-remyelinating effect by modulating OPC–neuron communication. Lastly, semaphorin 3F favors remyelination by promoting OPC recruitment to demyelinating lesions. This molecule has been found in MS tissue, specifically in microglia from active lesions, as opposed to chronic plaques and noninflammatory lesions [159]. This indicates that semaphorin 3F is present in those lesions prone to remyelination. As discussed above, there is no well-characterized pro- or anti-inflammatory environment in MS lesions. Interestingly, some cytokines secreted by activated microglia/macrophages, and classically considered to be pro-inflammatory, have also been implicated in myelin repair in animal models of MS. One example is TNF-α in the cuprizone model, which promotes OPC proliferation and remyelination via the TNF receptor 2 (TNFR2) [164]. Another cytokine that is important for OPC differentiation and myelin restoration is IL-1β, acting through the induction of IGF-1. Thus, in the cuprizone model, an increase in IL-1β was associated with IGF-1 accumulation and increased numbers of microglia/macrophages in WT mice, while IL-1β^−/−^ mice have weaker IGF-1 mRNA expression, less OL maturation, a decrease in neuron-glia antigen 2 (NG2)^+^ OPC accumulation, and fewer remyelinated axons [165].

In addition, the alternatively activated macrophage secreted enzyme interleukin-4 induced 1 (IL4I1) indirectly enhances remyelination in the LPC-induced demyelination model by controlling T cell-driven inflammation. In this case, focal demyelination was caused by stereotaxic injection of LPC in the ventral funiculus of the SC. The densities of pro-inflammatory macrophages (CD11b^+^INOS^+^) and axonal injury are enhanced in IL4Rα^−/−^ demyelinated mice, in conjunction with deficient remyelination of SC lesions, effects that were rescued by the focal administration of recombinant IL4I1 [166].

Finally, microglia/macrophages also participate in the degradation of some components of the extracellular matrix that accumulate after injury, molecules that inhibit OPC differentiation and, hence, remyelination (Figure 3). One example is the matrix metalloproteinase-9 (MMP9) secreted by microglia and macrophages, which promotes remyelination after LPC injection by processing the chondroitin sulfate proteoglycan NG2, thereby rescuing OL maturation [150]. Fibronectin aggregates in chronic MS lesions, preventing OPC differentiation and, therefore, remyelination [167]. MMP7 cleaves fibronectin aggregates, and its proenzyme proMMP7, which is mostly produced by alternative (IL-4)-activated microglia and macrophages, is expressed poorly in chronic MS lesions relative to shadow plaques, in part explaining the failure of remyelination in these lesions [151].

Insufficient efforts have been made regarding the analysis of the effect of DMTs on immune CNS resident cells. In general terms, it has been shown that DMTs modulate microglia activation towards an alternatively activated activity state, which is related to the increase in the release of anti-inflammatory cytokines. Whether these modifications have a direct impact on the promotion of myelin repair deserves further investigation [27,168]. 

### 4.2. T Cells

Several studies have highlighted the beneficial role of inflammation in remyelination, involving cells relevant to both the innate and adaptive immune response in the process. In this sense, T cells, B cells, and plasma cells were found in newly forming MS lesions, together with differentiated OLs, suggesting that the adaptive immune response could positively influence OL regeneration and myelin repair [169]. In particular, T cells emerged as important players in myelin regeneration, fulfilling both positive and negative roles [170]. In fact, CD4^+^ T cells from MS patients injected into LPC-induced demyelinated mice affected the myelin repair process distinctly, as diverse patterns of remyelination were seen in these mice [171]. Thus, some of these MS lymphocytes had a beneficial role in remyelination, while others had a detrimental effect, as observed in MS patients. Interestingly, in vitro experiments revealed that MS lymphocytes drive microglial cells towards a more pro-inflammatory state (iNOS^+^) than those isolated from HC, enhancing OPC proliferation yet preventing their differentiation. Moreover, lymphocytes from MS patients have a different secretory profile from those isolated from HC, with increased levels of IL-7 and IL-20 and decreased levels of the chemokine CCL19.

In an early study, Rag-1-deficient mice lacking both B and T cells presented impaired remyelination after LPC-induced demyelination. Moreover, two different groups of mice with CD4^+^ or CD8^+^ T cell depletion also exhibited reduced remyelination, meaning that both cell types are required for this process [172]. Subsequently, the detrimental role of pro-inflammatory Th1 and Th17 cells in remyelination was demonstrated in a model combining cuprizone-mediated demyelination with CD4^+^ T cell transfer from EAE mice [173]. By contrast, decreased CD4^+^ T cell infiltration in an MS model generated with the mouse hepatitis virus and transplanted with human neural precursor cells derived from human embryonic stem cells resulted in reduced remyelination. Interestingly, high levels of FoxP3^+^ Treg were found in peripheral lymph nodes but not in the CNS in this model [174], suggesting that this constitutes the T cell subset implicated in myelin regeneration. Thus, lower levels of FoxP3^+^ Treg in the CNS would be associated with impaired remyelination. This observation was later corroborated in FoxP3-DTR transgenic mice in which diphtheria toxin administration causes FoxP3^−^ Treg depletion [17], producing defects in OL differentiation and remyelination after focally induced demyelination in the SC that could be rescued by Treg injection. The relevance of Treg in this process was also proven in the brain of the cuprizone administered mice, as well as in organotypic brain slice cultures demyelinated with LPC in vitro, where addition of Treg-conditioned media increased the number of mature OLs, the myelination index, and the number of remyelinated axons. Interestingly, protein profiling of this conditioned media identified CCN3 to be the secreted factor that promoted OL differentiation both in vitro and ex vivo (Figure 3) [17]. Furthermore, a very recent study demonstrated Treg expansion following human neural stem cell transplantation that promoted remyelination in the SC of EAE mice, corroborating the need for Treg to successfully promote remyelination [175].

### 4.3. B Cells

Among the different roles proposed for B cells in the context of MS, there is growing evidence of their participation in remyelination [176]. As mentioned above, B cells have been implicated in OL and myelin regeneration due to their presence in newly forming MS lesions, together with differentiated OLs [169]. B cells influence remyelination through both antibody production and the secretion of specific cytokines. In terms of immunoglobulins, they can adopt pathogenic or reparative roles depending on their microenvironment [4]. Classically, some autoantibodies from the sera of MS patients and EAE mice have been considered deleterious for myelin due to their proteolytic activity towards MBP [177], although other anti-OL antibodies favor remyelination. Thus, two human monoclonal antibodies, sHIgM22 and sHIgM46, have been identified that are directed against OL surface antigens and that induce remyelination in both the TMEV [178,179] and LPC models [180]. The possible mechanism for IgM-mediated remyelination points to OPC protection from apoptosis rather than the promotion of OPC differentiation, as demonstrated in vitro (Figure 3) [181].

Finally, while classically considered to be dangerous, B cell-released cytokines may also be beneficial in remyelination. Transfusion of IL-10^+^ Breg in the EAE model promotes the expansion of CNS-infiltrating IL-10^+^ T cells, subsequently ameliorating the EAE clinical course and increasing remyelination in the SC. After Breg transfer, the thickness of myelin around remyelinated axons increases, and OPCs and newly-generated OLs are recruited [182]. Therefore, Breg could favor remyelination through IL-10 production.

### 4.4. Myeloid Derived-Suppressor Cells (MDSCs)

Although there are abundant data regarding the role of M-MDSCs (CD11b^+^Ly-6G^−/lo^Ly-6C^hi^) in ameliorating the clinical course of EAE [102,103,106,111,183] and TMEV-IDD [108] by suppressing T cell activity, their implication in remyelination has been explored less intensely. In this regard, we have shown a clear inverse correlation between the abundance of splenic M-MDSCs and the degree of demyelination within the SC of EAE mice [104]. We also found that the higher density of M-MDSCs in demyelinated SC lesions of EAE mice is correlated with a higher density of OPCs (NG2^+^ cells) in the adjacent periplaque. Moreover, the putative role of M-MDSCs as facilitators of myelin repair has been reinforced since M-MDSC-conditioned media promotes OPC survival, proliferation, differentiation, and remyelination. Interestingly, one of the relevant soluble factors in this conditioned medium was seen to be osteopontin (Figure 3) [184]. Nevertheless, further studies will be required to address the direct effect of M-MDSCs on remyelination in vivo or in the real context of the CNS of MS patients. Moreover, there are still no data on the role of PMN-MDSCs in remyelination.

## 5. Concluding Remarks

In MS and EAE, there are several immune cells that might be among the principal pathological orchestrators of MS, where the failure to fully resolve inflammation participates in chronic inflammation. The resolution of inflammation is the result of complex interactions between peripheral and resident CNS cells. In this review, we highlight the information available regarding the role of immune regulatory cells in the maintenance of tolerance and remyelination in MS. The data regarding peripheral immune regulatory cells are controversial, but overall, these cells seem to be impaired or altered in MS patients. We also reveal the complex relationship between the regulatory cells located at the periphery and those at sites of CNS inflammation, suggesting that future studies should better analyze the regulatory cells in the CNS to shed further light on their role in MS pathogenesis. The literature offers controversial results regarding the role of regulatory immune cells in MS, probably due to the use of different protocols, the wide variety of phenotypes to define regulatory cells, and the variation in patient cohorts and treatments. In this sense, better understanding of endogenous mechanisms involved in the resolution of inflammation might lead to novel therapeutic strategies targeting regulatory cells. However, these data have been largely overlooked, and the establishment of definitive research protocols remains to be established. Currently, different therapies with immunomodulatory actions and the potential to re-establish immune homeostasis could be beneficial in MS disease. In conclusion, the immune mechanisms involved in the resolution of peripheral inflammation and in the promotion of remyelination in MS must be considered in conjunction with opening the door to future treatment strategies that effectively induce peripheral and CNS tolerance, contributing to more complete MS resolution.

## Data Availability

Not applicable.
